# Endoscopic Diagnostic System Using Autofluorescence

**DOI:** 10.1155/DTE.5.59

**Published:** 1999

**Authors:** S. Takehana, M. Kaneko, H. Mizuno

**Affiliations:** 1 Product Development Department Endoscope Division Olympus Optical Co., Ltd. 2951 Ishikawa-cho Hachioji-shi Tokyo 192-8507 Japan

## Abstract

A fluorescence imaging system (Xillix LIFE – Lung Fluorescence Endoscopy system) using fluorescence for the accurate diagnosis and early detection of lesions through an endosocope has been developed. This system has applied an optical diagnostic technology to functionally diagnose lesions which have been difficult to morphologically recognize or are occult with conventional endoscope. The benefit of this system in the diagnosis of lung cancer has already been confirmed in the US and Japan, and feasibility of the system in the gastric intestinal field has also been evaluated.


*Diagnostic and Therapeutic Endoscopy*, Vol. 5, pp. 59–63
Reprints available directly from the publisher
Photocopying permitted by license only

© 1999 OPA (Overseas Publishers Association) N.V.
Published by license under
the Harwood Academic Publishers imprint,
part of The Gordon and Breach Publishing Group.
Printed in Singapore.

### Endoscopic Diagnostic System Using Autofluorescence

S. TAKEHANA*, M. KANEKO and H. MIZUNO

*Product Development Department Endoscope Division, Olympus Optical Co., Ltd., 2951 Ishikawa-cho Hachioji-shi, Tokyo, 192-8507 Japan*

**A fluorescence imaging system (Xillix LIFE – Lung Fluorescence Endoscopy system) using fluorescence for the accurate diagnosis and early detection of lesions through an endosocope has been developed. This system has applied an optical diagnostic technology to functionally diagnose lesions which have been difficult to morphologically recognize or are occult with conventional endoscope. The benefit of this system in the diagnosis of lung cancer has already been confirmed in the US and Japan, and feasibility of the system in the gastric intestinal field has also been evaluated.**

*Keywords:* Autofluorescence, Early detection, Fluorescence endoscope, Spectroscopic technology

### INTRODUCTION

By adopting spectroscopic technologies such as fluorescence of light, absorption of light, and Raman-scattered light, abnormalities of living tissue can be detected on the cellular or macroscopic level. Recently, a new technology, optical diagnosis, has been calling attention that applies the above spectroscopic technologies to the "living body as it is" to functionally diagnose lesions that have been difficult to be determined by conventional morphological diagnosis [1–4]. Particularly, research and development of "fluorescence imaging" and "optical biopsy" have been actively implemented: fluorescence imaging has been developed in view of early detection and diagnosis of lesions, that have been difficult to detect with conventional endoscopy. This has been done by means of displaying *in vivo* video images endoscopically using autofluorescence emitted from living tissue. Emitted autofluorescence is a result of when a light of specific wavelength (light between UV and blue region) excites tissue. This can be done without using drugs such as photosensitizing substances [5–7]. Optical biopsy has been developed to provide pathological diagnosis with an optical fiber inserted through an endoscope channel, in place of biopsy [8,9]. These autofluorescence technologies are expected to be established in the near future as a new diagnosing method in conjunction with an endoscopic fluorescence imaging endoscopes.

As stated above, development of an endoscopic fluorescence imaging system has been conducted to endoscopically display image of autofluorescence

* Corresponding author.

to provide new diagnostic form by exploiting the difference between the spectrums of normal and abnormal tissue [10,11].

### MATERIALS AND METHODS

### Overview of the Device

The LIFE-Lung system was first developed in the world by Xillix Technologies Corp., Canada, in 1990, and the system was found to be useful in detecting early lesion [10–12]. This system has received FDA approval in 1996. Olympus Optical Co., Ltd. started selling the product worldwide in 1997. The outlook appearance of the system is shown in Fig. 1.

### System Components

Components of this system are shown in Fig. 2. This system is comprised of an Illumination Console of He–Cd laser (442 nm) which produces blue light, a LIFE camera which includes ICCDs, high-sensitivity imaging devices, that detect subtle changes in autofluorescence, an Imaging Console which processes and displays the autofluorescence images, and a commercially available endoscope system.

An excitation light source of 442 nm wavelength produces blue light, delivers to the tissue surface through an endosope. Then, the low light level autofluorescence emitted from the tissue is amplified through an endosope. Then, the low light level autofluorescence emitted from the tissue is amplified by 5,000 to 10,000 times and detected by the LIFE camera attached to the eyepiece of the endoscope. This LIFE camera installs two high-sensitivity CCDs that capture green and red autofluorescence, and those captured image signals are, respectively, processed by the image processing unit in real-time, and then displayed on the monitor as a color image (Fig. 2) (Table I).

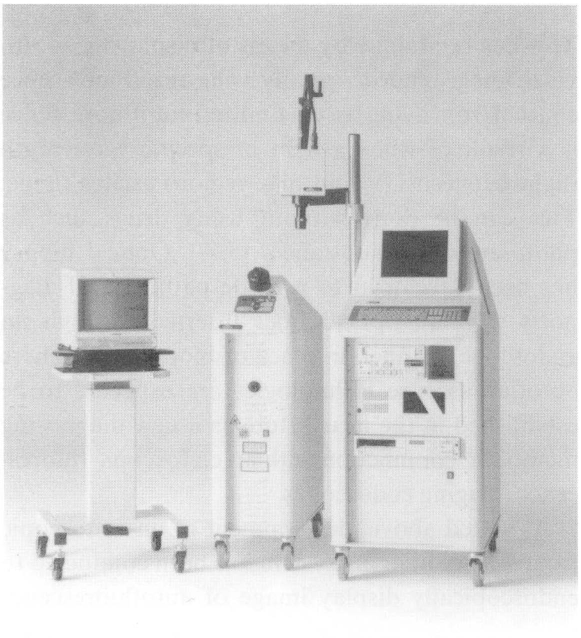

FIGURE 1 Outlook appearance of the system.

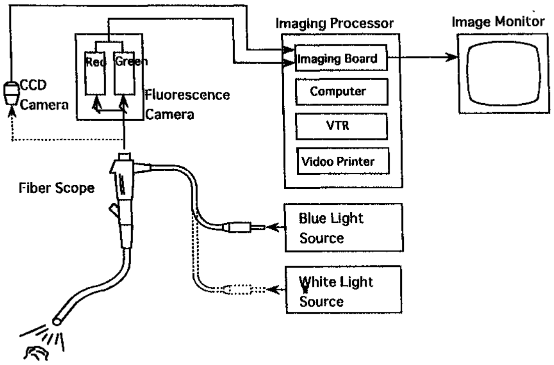

FIGURE 2 Schematic diagram of endoscopic fluorescence system.

TABLE I Specifications of this system

| | |
|---|---|
| *Illumination console* | |
| Laser | He–Cd laser |
| Wavelength | 442 nm |
| Light output | 15 ~ 30 mW |
| *LIFE camera* | |
| Imaging device | ICCD |
| Video I/O | RGB signal |
| *Imaging console* | |
| Image management | Digital filing<br>SVHS video cassette recorder<br>Video printer |

### Specifications and Features of the System

Specifications of this system is shown in the Table I. As stated in the above, this system does not require administration of drugs such as photosensitized substances for the early detection and diagnosis of lesion, thus there is no need to anticipate side-effects. Also, the laser output of this system is kept low and its safety with regard to bioeffects has been confirmed. Therefore, this system can be used just as with conventional white-light bronchoscopy.

### Principle of Autofluorescence

Fluorescence imaging is designed for the early detection and diagnosis of lesions by endoscopically illuminating light against tissue, processing the emitted fluorescence into images, and displaying such lesions that have been difficult to detect or diagnose under conventional white-light endoscopy as the difference of fluorescence intensity or color tone.

Figure 3 is the result of autofluorescence spectrum measured of normal and abnormal tissue when blue light was illuminated to those living tissues through an optical fiber inserted into the endoscope channel [5,6]. Figure 3 shows autofluorescence spectrum of normal bronchial tissue and CIS (Carcinoma *in situ*) using an excitation wavelength of 442 nm (He–Cd laser). While in normal tissue, the shape of fluorescence spectrum demonstrated its peak in green with gradual decrease at longer wavelength, the shape of overall spectrum in abnormal tissue was rather flat with decreased spectrum centering to the short wavelength side and with a small rise in red side.

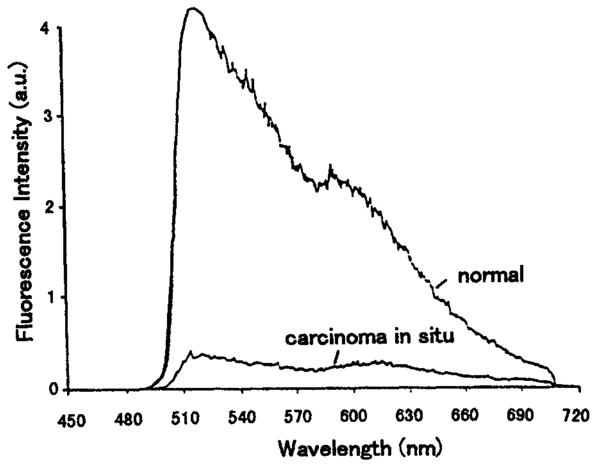

FIGURE 3 Autofluorescence spectrum in normal and abnormal tissue in the bronchus.

Although the precise mechanism of different autofluorescence spectrums detected in normal and cancerous tissue has not been established, the following has been considered: It has been considered highly possible that collagen, flavoprotein, NADH, and porphyrin, known as autofluorescence substances existing in the living tissue, have something to do with the different spectrums as has the tissue architecture and the blood content.

With this LIFE-Lung, it has been considered that the system observes the following differences based on the wavelength excited/detected from the above substances.

(1) Cancerous tissue has higher metabolism than normal tissue and therefore, its blood volume increases while oxygen concentration in the cells decreases. Because of the increase of blood volume and accumulation character which is specific to cancer, the amount of porphyrin is increased (red fluorescence is increased), and at the same time, flavin is reduced (green fluorescence is decreased) as oxygen conoentration is decreased.

(2) Autofluorescnece is intensely produced from submucosa stroma (ex. collagen), but epithelium, mucosa, and cancerous tissue emit very little fluorescence. Because of thicker epithelium and mucosa in cancerous region than in normal region and of the presence of cancer, fluorescence of green region is intensely absorbed. (Tissue permeability of light is higher in red region than in green region: 100–1000 times greater per cm.)

### RESULTS

### Clinical Applications

A Multi-Center Study LC01 conducted from 1994–1995 in North America reports that in 173 patients, detection ratio for moderate/severe dysplasia or greater was improved from 24.6% (with LIFE-Lung compared to that with conventional white-light endoscopy alone) to 66.9%, and that their specificity were 92.0% and 70.2%, respectively, without significant difference.

In Japan as well, Yokomise *et al.* and Ikeda *et al.* conducted a study on the benefits of the LIFE-Lung from 1995 fall to 1996 spring [13,14]. Yokomise *et al.* and Ikeda *et al.* each conducted a comparative study on 30 patients with existing or suspected lung cancer comparing with the result of conventional white-light bronchoscopy and both admitted effectiveness of LIFE-Lung. Yokomise *et al.* reported that compared to conventional white-light bronchoscopy alone, detection ratio of metaplasia and cancer was improved from 65% to 90%, and that specificity was improved from 71% to 77.4% by using autofluorescence bronchoscopy jointly with conventional bronchoscopy. Ikeda *et al.* also report that detection ratio for metaplasia was improved from 28% to 96% by the joint use of autofluorescence bronchoscopy with conventional bronchoscopy than by the latter only. Since a screening test using sputum cytology has been carried out particularly in Japan, the LIFE-Lung is expected to serve in diagnosis of early lung cancer in subjects who have turned out to be positive in the sputum cytology.

**Application to GI**

Figure 4 shows autofluorescence spectrum of normal and abnormal (precancerous) tissue when the light of 437 nm (a combination of high-pressure Halogen lamp and a blue band-pass filter) was emitted into the esophagus. Autofluorescence spectrum similar to that in the bronchi is shown. Based on this fact, Olympus Optical Co., Ltd. and Xillix Technologies Corp. has jointly been developing a fluorescence endoscopy system for gastric intestinal tract (LIFE-GI imaging system) based on LIFE-Lung.

In the gastrointestinal tracts such as stomach and colon where body cavity is larger than in bronchi and whose mucosa contains a lot of capillary blood vessels, good-quality fluorescence image cannot be acquired with the system designed for bronchi due to the shortage of excited light intensity. Therefore, by combining mercury lamp and blue-exiting filter to blue light source, twice as much intensity of light as that of He–Cd laser can be obtained at the tip of the endoscope, which enabled autofluorecence observation in the large body cavity of gastrointestinal tracts. By using a device in which this blue light source is installed, we have applied this technology to the esophagus, stomach, and colon [6,15–25].

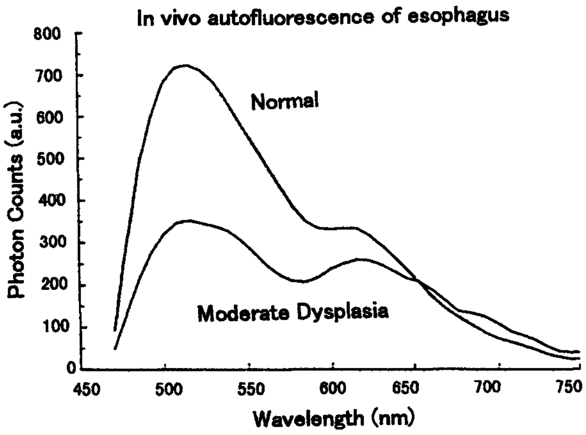

FIGURE 4 Autofluorescence . spectrum in normal and abnormal tissue in the esophagus.

Watanabe *et al.* applied this device in patients (total 12 lesions) who were suspected of remnant after the endoscopic treatment for stomach cancer, and reported that they were able to identify the presence and location of remnant in 3 cases by biopsies performed under fluorescence observation that they were not able to recognize under conventional white-light endoscopy [24]. Also, Yano *et al.* applied this device to 47 patients with stomach cancer and reported that they have succeeded in finding second-lesion in one case which were not possible to indicate under conventional white-light endoscopy [25]. Based on these past clinical trials, feasibility of LIFE-GI in detecting lesions that have been difficult to recognize with conventional white-light endoscopy has been suggested. It is necessary in the future to further increase the number of cases and clarify clinical benefits.

### DISCUSSION

Various studies have long been carried out in an attempt to diagnose cancer by using fluorescence, but observing the autofluorescence was especially difficult due to its weak intensity. However, recent improvement in illumination and in high-sensitivity camera has enabled real-time display and observation of even subtle changes in autofluorescence endoscopically. The technology was developed and put into practice using the technology developed in the LIFE-Lung system.

The past clinical findings suggests the feasibility of autofluorescence endoscopy to become an effective means of diagnosis for the detection and localization of precancerous tissue such as micro cancer and metaplasia in the bronchi and gastrointestinal tracts. We are also expecting that in the future, combination of this technology for early diagnosis and minimal invasive treatment such as endoscopic treatment and/or PDT will increase opportunity for patients to undergo high QOL treatment of minimally invasive.

### *References*

[1] Tamura, M. *et al.* Hikari ga hiraku igaku iryou no sinseiki (in Japanese). This is Medicine in Japan 1995; **242**: 24–26.

[2] Bohorfoush, A.G. Tissue spectroscopy for gastrointestinal diseases. *Endoscopy* 1996; **28**: 372–380.

[3] *SPIE*, **2387–2388**, 1995.

[4] Hung, J. Detection and localization of pre-cancerous lesions and early lung cancer using tissue autofluorescence. Doctor of philosophy Thesis, University of British Colombia, Dept. of physics. Canada, 1992.

[5] Palcic, B., Lam, S., Hung, J. *et al.* Detection and localization of early lung cancer by imaging techniques. *Chest* 1991; **99**: 742–743.

[6] Zeng, H., Weiss, A., MacAulay, C. *et al.* Development of a fluorescence video endoscopy imaging system for the early detection of cancer in the gastrointestinal tract. *SPIE* 1997; **2976**; 291–296.

[7] Wang, T., Dam, J., Crawford, J. *et al.* Fluorescence endoscopic imaging of human colonic adenomas. *Gastroenterology* 1996; **111**(5): 1182–1191.

[8] Cothren, R., Sivak, M., Dam, J. *et al.* Detection of dysplasia at colonoscopy using laser-induced fluorescence: a blinded study. *Gastrointestinal Endoscopy* 1996; **44**: 168–176.

[9] Vo-Dinh, T. *et al. Lasers in Surgery and Medicine* 1995; **16**: 41–47.

[10] Lam, S., Hung, J., MacAulay, C. *et al.* Fluorescence imaging of early lung cancer. *LEEE Engineering Medicine and Biology Society* 1990; **12**(1): 1142–1143.

[11] Palcic, B., Jaggi, B., Pon, A. *et al.* Development of a lung imaging fluorescence endoscope. *IEEE Engineering Medicine and Biology Society* 1990; **12**(1): 196–197.

[12] Lam, S., MacAulay, C., Hung, J. *et al.* Detection of dysplasia and carcinoma *in situ* with a lung imaging fluorescence endoscope device. *J. Thorac. Cardiovasc. Surg.* 1993; **105**: 1035–1040.

[13] Yokomise, H., Yanagihara, K., Fukuse, T. *et al.* Clinical experience with Lung-Imaging Fluorescence Endoscope (LIFE) in patients with lung cancer. *J. Bronchology* 1997; **4**(3): 205–208.

[14] Ikeda, N., Kim, K., Okunaka, T. *et al.* Early localization of bronchogenic cancerous/precancerous lesions with lung imaging fluorescence endoscope. *Diagnostic and Therapeutic Endoscopy* 1997; **3**:197–201.

[15] Ogihara, T., Watanabe, H., Namihisa, A. *et al.* Aratani kaihatsu sareta naisikyou teki keikou gazou kansatsu souti no siyou keiken (in Japanese). *Gastroenterological Endoscopy* 1996; **37**(Suppl. 2): 1879.

[16] Namihisa, A., Watanabe, H., Miwa, H. *et al.* Naishikyou teki keikou gazou kansatsu souti ni yoru ibyouhen kansatsu no yuuyou sei ni kansuru kentou (in Japanese). *Gastroenterological Endoscopy* 1996; **37**(Suppl. 2): 1914.

[17] Yano, H., Iishi, H. and Tatsuta, M. Diagnosis of early gastric cancers by endoscopic autofluorescence imaging system. *7th Asian-Pacific Congress of Digestive Endoscopy* 1996; 122.

[18] Watanabe, H., Ogihara, T., Namihisa, A. *et al.* Application of a new fluorescence endoscopic imaging system to the detection of small remnant of endoscopically resected gastric. *7th Asian-Pacific Congress of Digestive Endoscopy* 1996; 124.

[19] Yano, H., Iishi, H. and Tatsuta, M. Diagnosis of early gastric cancers by an endoscopic autofluorescence imaging system. *Endoscopy* 1996; **28**: 898. (*5th United European Gastroenterology Week*)

[20] Lilge, L., DaCosta, R., Scheider, D. *et al.* Laser induced fluorescence spectroscopy at endoscopy. *Endoscopy* 1996; **28**: 1168. (*5th United European Gastroenterology Week*)

[21] Ierland, M. and Tytgat, G. Detection of dysplasia using the XILLIX-Life-GE-System. *Endoscopy* 1996; **28**: 1169. (*5th United European Gastroenterology Week*)

[22] DuVall, G., Kost, J., Schleider, D. *et al.* Laser induced fluorescence (LIF) endoscopy (E): A pilot study of a real-time (RT) autofluorescence imaging system for early detection of dysplasia and carcinoma in the gastrointestinal (GI) tract. *Endoscopy* 1996; **28**: 1170. (*5th United European Gastroenterology Week*)

[23] Watanabe, H., Ogihara, T., Namihisa, A. *et al.* Usefulness of a new auto-fluorescence endoscopic imaging system for the diagnosis of gastric neoplasm. *Endoscopy* 1996; **28**: 1589. (*5th United European Gastroenterology Week*)

[24] Watanabe, H. and Sato, N., Bishou igan sindan ni taisuru naisikyou teki riaru taimu jika keikou gazou no yuuyou sei (in Japanese). *Gastroenterological Endoscopy* 1997; **39**(Suppl. 1): S16-9.

[25] Yano, H., Iishi, H. and Tatsuta, M. Diagnosis of early cancers by an endoscopic autofluorecence imaging system. *Gastrointestinal Endoscopy* 1997; **45**(4): 314.

